# Early incident and subsyndromal delirium in older patients undergoing elective surgical procedures: a randomized clinical trial of an avoid delirium protocol

**DOI:** 10.3389/fanes.2023.1268263

**Published:** 2023-11-02

**Authors:** Alisha Sachdev, Yabtsega Moges, Micah Rubin, Amanda C. Sremac, Zoe Arvanitakis, Robert J. McCarthy

**Affiliations:** 1 Department of Anesthesiology, Rush University Medical Center, Chicago, IL, United States; 2 Rush Alzheimer’s Disease Center, Rush University Medical Center, Chicago, IL, United States

**Keywords:** delirium, subsyndromal delirium, benzodiazepine, anesthesia, postoperative care unit

## Abstract

**Background:**

Pharmacological avoidance guidelines for preventing delirium have been suggested; however, there are limited pragmatic studies of these strategies. Early (<24 h) delirium can be observed in the postoperative care unit and is associated with an increased risk of subsequent delirium. We examined the effectiveness of an avoid delirium protocol (ADP) in older (>65 years) patients undergoing elective surgeries.

**Methods:**

The randomized controlled trial assessed an ADP developed using the American Geriatric Society’s Clinical Practice Guidelines for Postoperative Delirium in Older Adults, on early (<24 h) incident or subsyndromal delirium. Delirium was assessed using the confusion assessment method before surgery, in the post-anesthesia care unit, and on postoperative day 1. The primary outcome of early delirium was the combined incidence of incident or subsyndromal delirium.

**Results:**

Early delirium was identified in 24/235 patients (10.2%) with a risk ratio of 1.27 (95% CI 0.59–2.73, *P* = 0.667) for patients randomized to the ADP. In cases with protocol adherence and no benzodiazepine use, early delirium was present in 10/ 73 (13.7%) compared to 14/148 (9.5%) in non-adherent cases [risk ratio 1.45 (95% CI 0.57–3.10, *P* = 0.362)]. Lower American Society of Anesthesiologists physical class [odds ratio 3.31 (95% CI 1.35–8.92, *P* = 0.008)] and an inpatient admission [odds ratio 2.67 (95% CI 1.55–4.87, *P* = 0.0002)] were associated with early delirium.

**Conclusions:**

Our findings suggest that pharmacological avoidance protocols limiting or avoiding the use of specific classes of medications are not effective in reducing early incident or subsyndromal delirium in older patients undergoing elective surgery.

## Introduction

Postoperative delirium (POD) presents acutely with confusion, disorientation, perceptual disturbances, emotional dysregulation, or sleep disturbances, manifesting within a few hours and peaking at 72 h following a surgical procedure (1). POD can be subdivided into three types, emergence, early, and late. Emergence delirium occurs during or immediately after emergence from general anesthesia, is associated with further episodes of delirium during hospitalization, and is reported to occur in up to 37.5% of older patients (2). Early delirium, within 24 h of surgery, frequently occurs in the post-anesthesia care unit (PACU) and is often associated with drugs administered intraoperatively (3). Assessments for early delirium can be successfully performed in the PACU using the confusion assessment method (CAM) after the patient reaches an Aldrete score of ≥9 (also known as the Post Anesthesia Recovery Score) (4). Early delirium in older patients has been reported to occur in up to 45% of patients receiving general anesthesia and is also associated with an increased risk for subsequent delirium during hospitalization (5). Late delirium can occur generally between postoperative days 2–5, and benzodiazepine drugs given in the postoperative period have been shown to increase the odds of its occurrence (6).

Subsyndromal delirium, defined as the presence of one or more of the core symptoms of delirium without reaching the threshold for a diagnosis of delirium, may represent a threshold state intermediate in severity between delirium and no delirium (7). Subsyndromal delirium shares similar risk factors with other forms of POD (8). The combined incidence of incident delirium and subsyndromal delirium in older patients using the CAM assessment has been reported to be between 19% and 54% following surgery in patients ≥65 years (9, 10).

**FIGURE 1:**
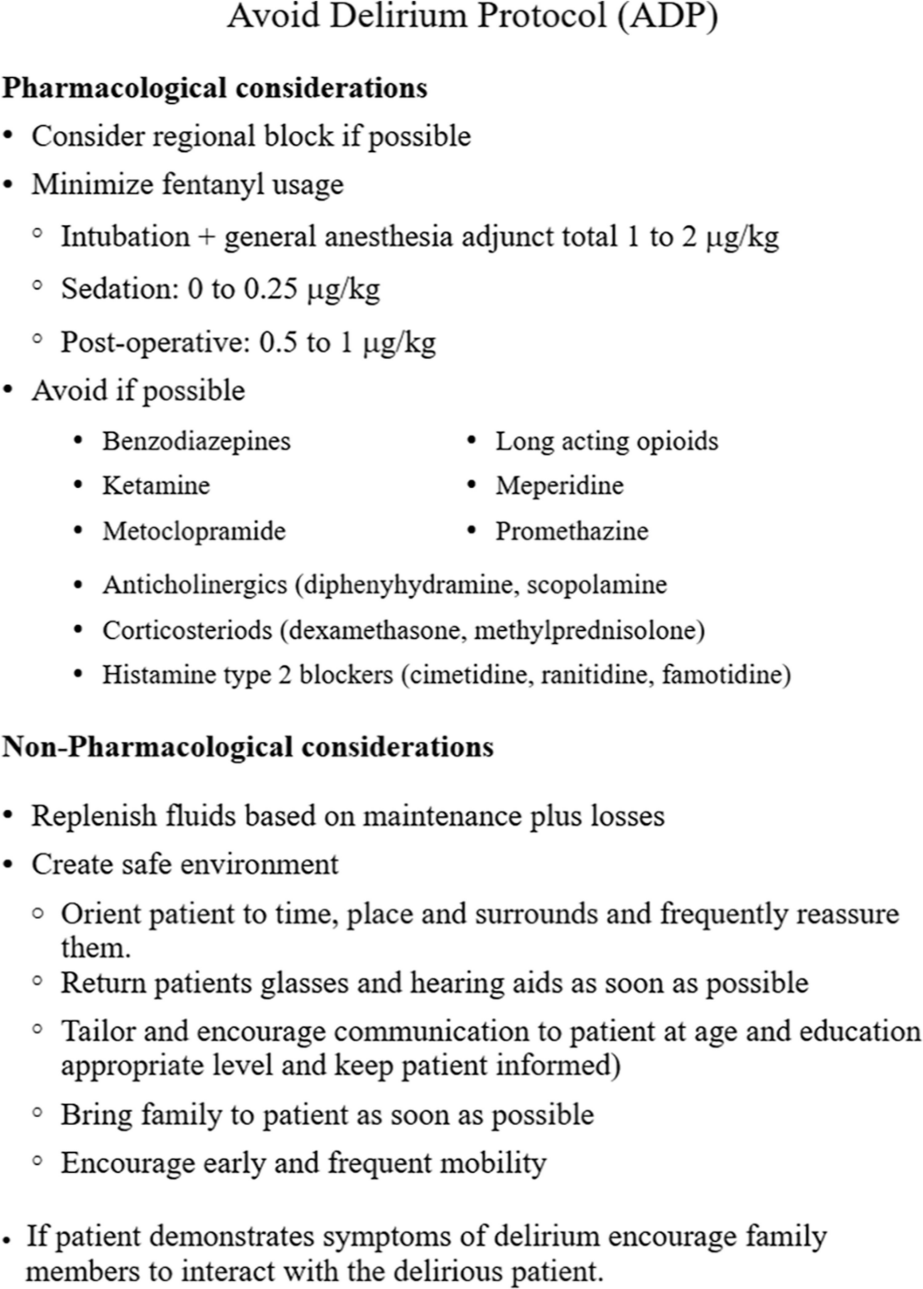
Avoid delirium protocol. Consort flow diagram. Based on the guidelines of the American Geriatrics Society Expert Panel on Postoperative Delirium in older adults.

Pharmacological and non-pharmacological interventions have been recommended to reduce the incidence of POD (11). Nevertheless, there are limited pragmatic effectiveness studies of these strategies (12). Non-pharmacological interventions that have been suggested include education and enriching the environment by adequately addressing needs of older adults, including vision and hearing aid availability, adequate pain control, and improved communication (13). Pharmacological classes that are recommended to be avoided include drugs with anticholinergic properties, corticosteroids, meperidine, sedative hypnotics, and polypharmacy (defined as ≥5 new medications) (14–16). Based on published guidelines from the American Geriatrics Society on avoidance of delirium in older patients, we developed an avoid delirium protocol (ADP) for use in the perioperative period (Figure 1). We hypothesized that the ADP would reduce the combined occurrence of early incident and subsyndromal delirium in older adult patients undergoing elective surgical procedures.

## Methods

This study was approved by the Institutional Review Board of Rush University (15062301-IRB01) and registered on ClinicalTrials.gov (NCT03541408, registration date 30 May 2018). This manuscript was prepared following CONSORT guidelines. The study was a randomized, controlled clinical trial of adults (>65 years) who underwent elective surgery between 1 May 2016 and 21 August 2020. Recruitment did not occur in March and April 2020 due to COVID restrictions. The intervention was the ADP developed for the prevention of delirium in older patients (11).

Eligible patients were scheduled to undergo an elective surgical procedure, ≥65 years of age, English speaking, and willing to comply with study activities. Patients currently in the hospital, undergoing cardiac surgery with bypass, open thoracotomy, neurosurgery, or with a history of stroke, psychiatric illness, or a diagnosis of dementia were excluded. Patients were identified 1–2 weeks prior to surgery and screened for eligibility. Patients were contacted by research personnel who explained the research protocol and obtained verbal consent to meet with the patient on the day of their procedure.

At the hospital, research personnel explained the study to eligible patients, who were given the consent form, allowed to review it, and ask questions regarding the study. Willing patients provided written informed consent. Following consent, subjects were randomized into either the ADP or standard of care arm by opening a sealed envelope containing the group allocation. Randomization was performed in blocks of 10 by a statistician not involved in the study using the SAS Proc Plan procedure assuming an equal probability group assignment.

Subjects were then assessed using the confusion assessment method for the intensive care unit (CAM-ICU) to ensure a negative test (no delirium) prior to surgery. They were also evaluated for cognitive function using the Mini Mental Status Examination (MMSE) (17). The MMSE tests cognitive function by examining orientation (10 points), registration (3 points), word recall (3 points), attention and calculation (5 points) language abilities (8 points), and visuospatial activity (1 point). A cutoff value of 24 or lower, out of a maximum of 30 points, was used to identify an abnormal test performance (18). Scores of the MMSE were not revealed to the subjects. Subjects with cognitive impairment (MMSE ≤24) were excluded from follow-up evaluations and data analysis. Study personnel then contacted and revealed the study group to the patient’s clinical care team. In addition, if the subject was randomized to the ADP group, a copy of the ADP protocol was sent to the care team.

The research team performing the MMSE and CAM-ICU scoring consisted of a physician (YM), and research assistants (MR and ACS) who were trained and supervised by a critical care anesthesiologist (AS) and a neurologist (ZA). Training included lectures, handouts, literature and videos, and one-to-one instruction at the bedside. The critical care anesthesiologist was contacted by research staff to discuss any cases where a clear assignment based on the scoring could not be obtained.

Subjects were also questioned regarding their work status and risk factors for delirium including: a history of delirium, functional independence, mobility devices used, history of falls, visual and/or hearing aid use, and physical trauma history. Comorbidities including renal and liver disease, history of depression, and number of current home medications were recorded. Hydration status was assessed by the clinical care team using clinical signs, capillary refill, skin turgor, and by examining the blood urea nitrogen and creatinine ratio. Research personnel recorded the status as hydrated or dehydrated in consultation with the clinical care team.

When the patient was transferred to the PACU, the research team was notified when the patient had achieved an Aldrete score of ≥9. The time from arrival to the recovery room until the first postoperative evaluation was recorded. The research evaluator determined the patient’s level of sedation using the Richmond Agitation and Sedation Score (RAAS). The RAAS scores are assessed on a 10-point scale from −5 to +4. Negative scores represent sedation, where −1 is drowsy and −5 is unarousable. A score of 0 is given for a calm and alert state (good recovery), positive +1 for restlessness, +2 and +3 for increasing agitation, and +4 for combative behavior (19). Subjects were required to have an RASS of −3 through +3 prior to delirium evaluation. The researcher also assessed the patient’s level of pain using the Numeric Pain Rating (NRS) scale, where 0 equals no pain and 10 equals the worst pain imaginable.

Delirium was then assessed using the CAM-ICU scoring system (20). The CAM-ICU was considered positive (delirium present) if the patient demonstrated an acute change in mental status or a fluctuation in mental status and inattention, plus either altered consciousness or disorganized thinking. Subsyndromal delirium was considered present if the subject displayed any of the CAM- ICU features but did not fulfill the diagnostic criteria for delirium. If no CAM-ICU features were present, the patient was considered to have no delirium. Patients who exhibited subsyndromal or incident delirium were assessed at least twice over the next 24 h using the RAAS scale so that the motoric subtype of delirium could be determined (21). At 24 h following surgery, subjects were again contracted either in person or via telephone by the researcher, to repeat the assessments. They also spoke with the subject’s companion or care giver regarding their impression of the subject’s health and mental status. They then asked the subject to rate their first night after surgery sleep on a scale of 0–10 where 0 was extremely not restful and 10 was extremely restful.

Data extracted from the medical record included age, sex, race, ethnicity, American Society of Anesthesiology Physical Status, medical history, surgical procedure, and admission status. Medications administered during and after surgery (up until discharge), duration of the surgery, total fluids administered intraoperatively, the presence, and duration of any hypotensive episodes (decrease in systolic blood pressure ≥30% of baseline). Intraoperative and recovery room opioids were converted to intravenous morphine equivalents (MME) (22).

Adherence to the ADP was assessed for all patients regardless of randomization group, by comparing medication administration in the medical record with the ADP and examining institutional enhanced recovery from anesthesia and surgery (ERAS) guidelines. Intraoperative administration of a benzodiazepine, meperidine, or morphine was considered a protocol violation, as was exceeding the recommended intubation and general anesthesia fentanyl doses. The use of corticosteroids, ketamine, anticholinergics, and histamine type 2 blockers were not considered protocol violations, when administered in accordance with ERAS guidelines for the specific surgical procedure. The subjects were classified as either protocol compliant or non- compliant.

### Statistical analysis

The primary outcome of the study was the occurrence of early delirium defined as CAM-ICU-assessed incident delirium or subsyndromal delirium in the first 24 h following surgery. The proportion of subjects demonstrating early delirium was compared between the ADP and control group by an intent-to- treat analysis, using a two-sided proportions test. As a sensitivity analysis, early delirium was compared between protocol compliant and non-compliant groups using the two-sided proportions test. Risk ratios and 95% confidence intervals were calculated using maximum likelihood estimations. Imbalances in subject characteristics, risk factors for delirium, admission, and surgery type between randomized groups and between protocol compliant and non-compliant groups were assessed by determining standardized difference (Hedges G for continuous data, and Cliff’s delta for ordinal and nominal data). Differences in operative and anesthesia data between groups were assessed using a chi-squared analysis for counts and the Mann–Whitney test for continuous data.

Secondary outcomes were times from PACU admission, from last drug administration of an agent ADP protocol, NRS pain score, and RAAS assessments at the time of CAM assessment. The motoric state of subjects that exhibited subsyndromal or incident delirium at 24 h was also compared. Times and pain scores were compared among groups using the Kruskal–Wallis *H*-test and RAAS scores using a chi-squared test.

An exploratory analysis was performed to assess risk factors for early delirium. Univariable differences in risk factors in subjects without delirium with those who had incident or subsyndromal delirium were compared using a chi-squared statistic for counts and the Mann–Whitney *U*-test for continuous data. Differences in binominal proportions and 95% confidence intervals were computed using the Clopper–Pearson method. Differences in medians and 95% confidence interval were determined using bootstrapping. Effect sizes of significant univariable binominal factors with early delirium are expressed as risk ratios and 95% CI determined using the Wald method. The effect size for continuous risk factors (intraoperative MME values, duration of surgery, intraoperative fluids, and postoperative pain in the first hour in the PACU) was determined for a random pair of values from a delirium subject from a no delirium subject as the Wilcoxon–Mann–Whitney odds and 95% CI.

A multi-variable adjusted estimate of the odds of early delirium was made using a logistic regression model using Firth’s bias reduction method. Firth and log-F-type penalized methods have been demonstrated to perform well in data with low prevalence outcomes without overfitting (23, 24). Variables that demonstrated a *P* < 0.2 on univariable analysis were entered into the model. The dependent variable was the presence or absence of incident or subsyndromal delirium. Independent variables entered the model were age; ASA physical status; visual impairment; functional dependence; admission type; type of surgery; type of anesthesia; administration of an opioid, dexamethasone, ondansetron, or midazolam; total intraoperative MME; total intraoperative i.v. fluids; estimated blood loss; duration of surgery; and NRS pain in the first hour in the PACU. Multicollinearity of the independent variables was assessed by evaluating the variance inflation factor (VIF) and the condition index (25). Variables exhibiting a VIF >5 were excluded from the analysis. A backward elimination with a significance level of 0.05 to stay in the model was performed to identify a parsimonious model with minimization of the Akaike information criteria (AIC0). Odds ratios are expressed as the exponent of beta with 95% confidence intervals of the estimates determined by penalized profile likelihood method (R package logistf).

Statistical analysis was performed using RStudio version 2023.06.1 build 524 (Posix Software, PBC, Boston, MA, USA; URL: http://www.posix.com/) and R version 4.3.1, release date 16 June 2023 (The R Foundation for Statistical Computing, Vienna, Austria).

We used published data to estimate the sample size, power, and other numbers needed for this study. In a study by Sharma et al. using methods similar to those used in this study, early delirium was detected in 44% of patients in the PACU following hip fracture repair (3). In contrast, Kanno et al. detected a combined incidence of early incident and subsyndromal delirium of 19% in a study of a mixed surgical population on the surgical ward soon after surgery (10). We assumed a conservative estimate of 25% of subjects would have incident or subsyndromal delirium and since no prior study has tested a comprehensive ADP, we estimated that a clinically important reduction in early delirium would be an absolute reduction by 10%. Group sample sizes of 250 per group would achieve 80% power to detect a difference of 10% in the proportion using a two-side *z*-test at an alpha of 0.05. We planned to enroll 530 subjects to account for subjects lost to exclusion following randomization. At approximately the midpoint of the study (*n* = 263), the observed rate of combined incident and subsyndromal delirium was 10.2% and a conditional power analysis with calculation of the futility index was undertaken (26). Sample size and power calculation were made using PASS 2008, release date 27 January 2011 [Power Analysis and Sample Size Software (2008). NCSS, LLC. Kaysville, UT, USA; ncss.com/software/pass].

## Results

**FIGURE 2:**
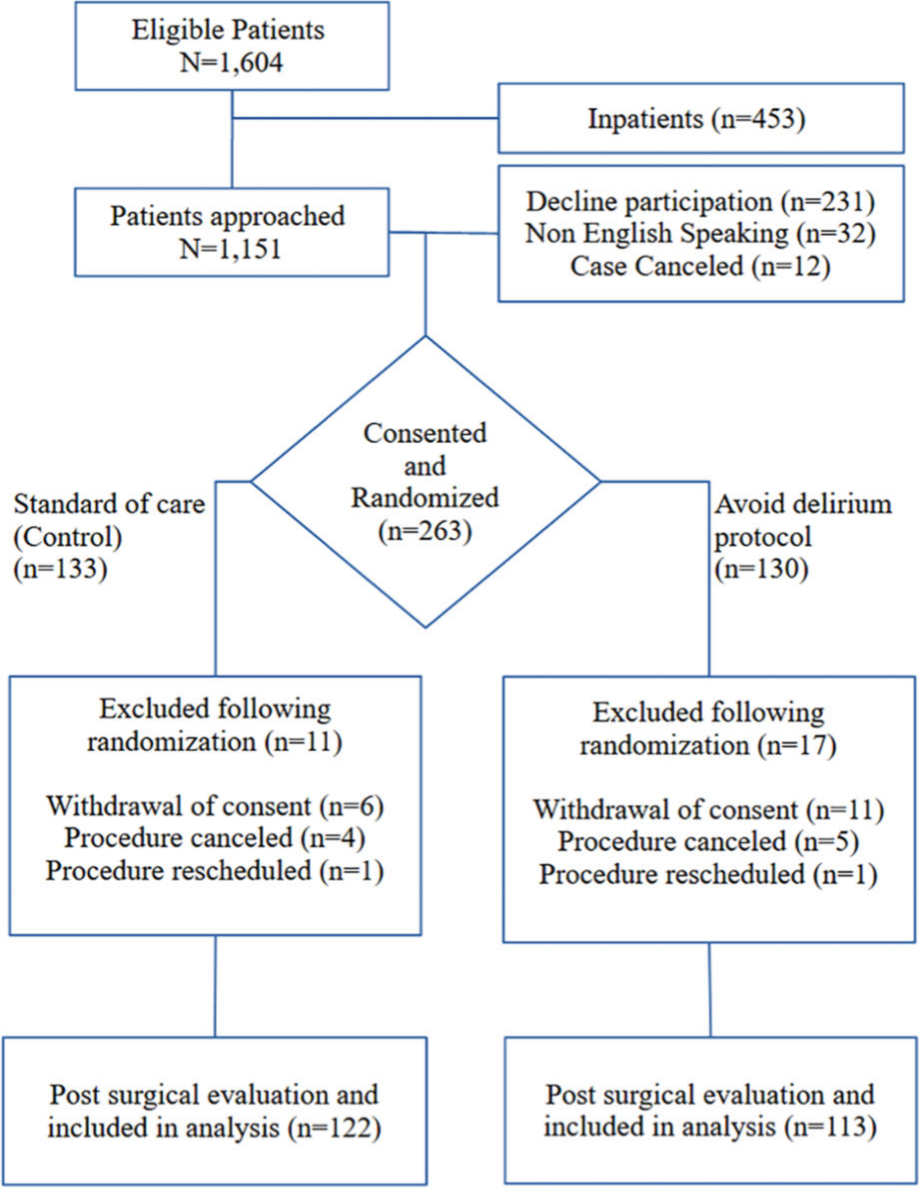
Consort flow diagram.

The flow of subjects in the study is shown in Figure 2. Of the 263/876 (31%) subjects consented, 28 were excluded following informed consent, 17 subjects withdrew consent, and 11 had cancelation or rescheduling of their procedure. Two hundred thirty-five subjects had their surgical procedure as scheduled, completed follow-up, and were included in the analysis. All subjects scored above 24 on the MMSE at screening, and no subject tested positive for incident or subsyndromal delirium pre- operatively.

Patient characteristics, risk factors, and types of surgery between the intent-to-treat as well as the per-protocol groups are shown in Table 1. Groups were well balanced with standardized differences <0.2 for all variables except for body mass index (BMI), and <0.1 for variables except work status, MMSE, and dehydration in the ADP and control groups. In the per-protocol groups, all variables had standardized differences <0.1 except for BMI and function dependence. Operative and anesthesia data between the intent-to-treat and per protocol groups are shown in Table 2. Dexamethasone and midazolam use was greater in the control group in both the intent-to-treat and per-protocol groups. Intraoperative MME use was greater in the intent-to-treat ADP group and the dose of vasopressor equivalents per kilogram was greater in the protocol adherent group compared to the non-protocol adherent control group.

The results of the delirium assessments in the first 24 h following surgery are shown in Table 3. In the PACU, incident delirium was identified in 6/235 (2.5%), and subsyndromal delirium was in 17/235 (7.2%) subjects. Over the 24-h period, 8/235 (3.4%) had incident delirium and 16/235 (6.8%) had subsyndromal delirium for an overall incidence of early delirium of 24/235 (10.2%). The median (first, third quartile) time from PACU admission to CAM assessment in subjects without delirium was 84 (53, 129) min and was increased by 24.5 (95% CI 5–43, *P* = 0.002) min in patients with early delirium. Early delirium patients primarily displayed a hypoactive motoric state in the first 24 h.

The incidence of early delirium was 13/113 (11.5%) in patients randomized to the ADP protocol, and 11/122 (9.0%) in those randomized to standard of care, with a risk ratio of 1.27 (95% CI 0.59–2.73, *P* = 0.667). In cases identified as protocol adherent, early delirium was present in 10/73 (13.7%) compared to 14/148 (9.5%) in subjects identified as not protocol adherent, with a risk ratio of 1.45 (95% CI 0.57–3.10, *P* = 0.362). Based on these findings the study was terminated at the 50% recruitment point, because using the estimated difference of 10% for the incidence early delirium in patient randomized to ADP, the probability of the study demonstrating a favorable outcome for the ADP with continuation to the intended sample of 500 was *P* < 0.0001 and the futility index was 1.0.

Univariable associations of subject characteristics, and operative and anesthesia factors with postoperative delirium, are shown in Table 4. Univariable differences in risk of early delirium were found for ASA class ≤2 compared with ASA class ≥3, general anesthesia compared with monitored anesthesia care or regional anesthesia, an inpatient admission compared with outpatient or 23-h stay, greater intraoperative MME use, greater intraoperative fluid administration; longer duration of surgery, and greater pain in the first hour in the PACU. Multivariable Firth logistic regression identified ASA class ≤2, odds ratio 3.31 (95% CI 1.35–8.92, *P* = 0.008) and an inpatient admission, odds ratio 2.67 (95% CI 1.55–4.87, *P* = 0.0002) as significant multivariable predicators of early delirium. Performance metrics of the parsimonious Firth model are likelihood ratio test = 18.76, df = 2, *P<* 0.001, AIC = 22.7, AUC = 0.76 (05% CI 0.68–0.84), and Brier score 0.08.

Postoperative nausea and vomiting requiring treatment in the PACU occurred in 7/210 (3.3%) no delirium and 4/24 (16.7%) early delirium subjects, risk ratio 5.0 (95% CI 1.5–15.8, *P* = 0.017). Median (first, third quartile) MME received in the post- anesthesia care unit was 1.7 (0–6.7) MME in the no delirium subjects, and 5.8 (0–12.7) MME in the early delirium subjects, difference of 4.1 MME, 95% CI −1 MME to 8.6 MME, *P* = 0.048. Median (first, third quartile) duration of stay in hours was 20.8 (2.1–30.5) in the no delirium subjects, and 46.9 (23.7–64.5) in the early delirium patients, difference 26.2 h, 95% CI 4.2 to 44.9 h, *P<* 0.001. Median (first to third quartile) rating of sleep quality on the first postoperative night was 8 (5–10) in the no delirium subjects and 5.5 (3–9) in the early delirium subjects, difference −2.5 (95% CI −3 to 0, *P* = 0.081).

## Discussion

The important finding of this study was the lack of efficacy of perioperative avoid delirium protocol based on minimizing or avoiding intraoperative drugs that have been associated with an increased in the risk of delirium. Despite a large body of evidence supporting various risk factors for developing postoperative delirium, no pragmatic studies exist to guide anesthetic management to attempt to prevent early delirium. Our study protocol was designed to minimize the use of agents that have been associated with postoperative delirium, including benzodiazepines, ketamine, and steroids. The protocol also incorporated non-pharmacological factors. Given that delirium can occur in patients with none of the known risk factors (27), the lack of effect of such a protocol has substantial clinical importance.

Prior studies excluding a single pharmacological class such an anticholinergic agent have not shown significant reduction in the incidence of delirium (28). Protocols involving non-pharmacological interventions including early mobilization, nutritional assistance, and cognitive exercises have demonstrated reduced delirium following gastrointestinal surgery, whereas non-pharmacological interventions coupled with a scheduled pain protocol reduced the duration of delirium but not the incidence in hip fracture patients (29, 30). Taken together, there is insufficient evidence to suggest that any single approach will benefit all patients in the intraoperative period, but rather judicious use of pharmacological agents coupled with non-pharmacological interventions is likely the best clinical approach.

We observed an incidence of early delirium (incident and subsyndromal) of (10.2%) older (≥65 years) subjects undergoing elective surgical procedures. The risk of early delirium was increased in patients with ASA score ≤2 compared with ASA score ≥3 and patients with hospital admission after surgery in our multi-variable analysis. Although univariable analysis found increased odds of receiving greater intraoperative opioid MMEs, more intravenous fluids, a longer duration of surgery, and greater pain in the first hour in the PACU compared with patient without delirium, these variables were not significant on multi- variable analysis. The lack of inclusion of these factors in the multi-variable analysis may reflect the small study sample size as well as the large number of factors that increase susceptibility to delirium. Early delirium subjects had an increased risk of nausea and vomiting in the recovery room, received more analgesia in the PACU, and had a longer duration of hospitalization. Our avoid delirium protocol, as designed, was not shown to be superior to the control arm using intent-to-treat or in our sensitivity per protocol analysis.

Prior studies have evaluated the incidence of early delirium and have reported an incidence between 11% and 45% (3, 5). Sharma et al. reported an incidence of 45% for early delirium in the recovery room following hip fracture surgery in patients aged 56–98 years, receiving general anesthesia using the CAM algorithm (3, 31). Radtke et al. reported an incidence of early delirium of 11% in adults (age >18) using the Nursing Delirium Screening Scale (Nu-DESC) in 862 subjects in the recovery room following elective general anesthesia (32). Neufeld et al. used physician raters who evaluated each patient using the Diagnostic and Statistical Manual of Mental Disorders (DSM)-IV criteria and observed a rate of early delirium of 45% (4). When compared in recovery room patients, the CAM-ICU method demonstrated equal specificity but less sensitive than the Nu-DESC or DSM-IV methods (33, 34).

Multiple studies have explored the potential risk factors for early delirium, occurring in the first 24 h after surgery. These risk factors can be broadly separated in two categories, predisposing risk factors and triggers that initiate the event (35). In addition to age >65, predisposing factors include clinical comorbidities, frailty, depression, functional disabilities, surgical risk, and preoperative cognitive dysfunction (36). We assessed preoperative cognitive status using the MMSE, but no subjects scored less than an MMSE score of 24 and there were only 2% of subjects with a value of <27, which has been suggested to be a cutoff value for mild cognitive impairment (37).

Types of surgery, surgical approach, and anesthesia have also been evaluated as risk factors associated with early delirium. Low operative stress procedures such as cataracts have a reported incidence of early delirium around 4%, whereas following high operative stress procedures such as vascular and cardiac surgery report incidences between 45% and 50% (38). We observed the highest incidence of early delirium (30%) in women undergoing gynecological procedures; however, there were only 10 cases in our study. In a study of postoperative delirium in older (>60 years) women (*n* = 103) undergoing surgery for suspected gynecologic malignancies, an incidence of delirium of 17.5% was observed (39). In the study, increased opioid administration, age, and preoperative medications were also associated with an increase in postoperative delirium. Surgical approach may also be an influencing factor, however, the study concluded that choice of anesthetic, general vs. regional, was more likely responsible for early delirium (40).

General anesthesia has been demonstrated to increase the risk of postoperative delirium compared with regional anesthesia (41). Anesthesia depth also has been demonstrated to impact the incidence of delirium, with lighter depth of general anesthesia reducing the incidence of delirium by 9% (odds ratio 0.58, 95% CI 0.38–0.88) (42). The use of regional anesthesia, an opioid reducing strategy, early mobilization, and the use of ERAS protocol were credited by Chew et al. for an incidence of delirium of 0% following 462 elective total joint surgeries at a single center (43). In the current study, 23% of surgeries were performed under regional anesthesia, more than 50% of the remaining procedures involved a regional anesthetic block as part of the perioperative management, and 67% of the uses of a restricted agent involved an ERAS protocol.

Preoperative fluid status and intraoperative factors such as hypotension and low cerebral oximetry values have been evaluated. Radtke et al. demonstrated that perioperative fluid fasting and choice of intraoperative opioid use may be modifiable risk factors for early postoperative delirium (28). Intraoperative hypotension has been associated with higher odds of postoperative delirium in critically ill patients (44). There is also evidence that blood pressure fluctuation, not hypotension or hypertension alone, may also be predictive of postoperative delirium (45). A meta-analysis of cerebral oximetry monitoring during surgery demonstrated a positive impact on postoperative neurocognitive function but not on delirium or postoperative stroke (46). We did not observe an association between preoperative dehydration or intraoperative hypotension >30% of baseline with early delirium.

The results of our study should only be interpreted in the context of its limitations. Our sample size was small, the study was performed at a single institution, and we terminated the study early because we observed statistical futility in changing our finding from the null hypothesis at the 50% accrual point. The small sample size also limits the certainty of our associations of the risk factors with the occurrence of delirium. We limited the surgical procedures to elective procedures of limited complexity and short duration because we primarily wanted to evaluate patients that are discharged soon after surgery and need to be ready for discharge quickly. The addition of longer and more complex procedures may have impacted our findings with respect to the incidence and risk factors associated with early delirium. We did not use intraoperative electroencephalography monitoring, so we cannot comment on the role of anesthesia depth on the occurrence of delirium.

We used the CAM-ICU method for detection of delirium in the recovery room. The CAM-ICU was originally developed for non-verbal patients in the intensive care unit, but due to its brevity and simplicity, the CAM-ICU is increasingly being used for detecting delirium in verbal patients outside the ICU setting (47). Nevertheless, the CAM-ICU method is less sensitive than the Nu-DESC and the 3D-CAM in verbal patients, and this may have contributed to an underestimation of the incidence of early delirium (32–34, 47).

We used the MMSE to assess cognitive function prior to surgery because it is easier to administer in the preoperative area than the Montreal Cognitive Assessment (MoCA); however, the MoCA is more sensitive to mild cognitive impairment (MCI), which may have allowed us to detect an effect of MCI in our cohort. Although our groups were generally balanced with respect to risk factors for delirium, adherence to ERAS regimens created many protocol violations that substantially reduced the number of subjects that had protocol adherence (29%). Because of the multidepartment nature of ERAS, implementation of avoidance protocols such as the ADP can be challenging, especially when ERAS adherence is an institutional metric.

In conclusion, the use of avoid pharmacological agents that have been associated with delirium coupled with non-pharmacologic patient centric care did not decrease the incidence of early delirium within the first 24 h following surgery. Our findings suggest that avoidance protocols are not likely superior to judicious use of pharmacological agents coupled with non-pharmacological interventions for minimizing early delirium.

## Figures and Tables

**TABLE 1:** Patient characteristics, risk factors, admission, and surgery type between patients randomized to the ADP and standard of care (control) groups, as well as the protocol compliant and non-compliant groups.

Number of subjects	Intent to treat			Per protocol^a^		
	Control	ADP	Standardized difference (95% CI)	Non-protocol adherent	Protocol adherent	Standardized difference (95% CI)
	122	113		162	73	
Age (years)	73.5 ± 6.0	72.5 ± 4.9	0.07 (−0.07 to 0.22)	72.9 ± 5.4	73.1 ± 5.8	−0.02 (−0.30 to 0.25)
Sex, *n* (%)						
Male	61 (50)	57 (50)	0.00 (−0.12 to 0.13)	84 (52)	34 (44)	−0.05 (−0.19 to 0.09)
Female	61 (50)	56 (50)		78 (48)	39 (56)	
Race/ethnicity *n* (%)						
Caucasian	94 (77)	90 (80)	0.03 (−0.08–0.13)	127 (78)	57 (78)	−0.01 (−0.13 to 0.10)
African American	17 (14)	15 (13)		26 (16)	7 (10)	
Hispanic	9 (7)	6 (5)		7 (4)	8 (11)	
Asian	2 (2)	2 (2)		3 (2)	1 (1)	
ASA physical status						
2	56 (46)	57 (50)	0.04 (−0.08 to 0.17)	76 (47)	36 (49)	0.02 (−0.11 to 0.16)
3	66 (54)	57 (50)		86 (53)	37 (51)	
BMI (kg/m^2^)	28.9 ± 6.4	30.7 ± 5.8	−0.30 (−0.56 to −0.05)	30.0 ± 6.2	29.2 ± 6.1	0.12 (−0.15 to 0.40)
Work status						
Retired	92 (75)	73 (65)	−0.11 (−0.22 to 0.01)	118 (73)	47 (64)	−0.08 (−0.21 to 0.05)
Working	30 (25)	40 (35)		44 (27)	26 (36)	
MMSE (0 to 30)	30 (29, 30)	30 (29, 30)	−0.18 (−0.52 to 01.5)	30 (29, 20)	30 (29, 30)	−0.04 (−0.39 to 0.20)
Risk factors						
History of delirium	2 (2)	1 (1)	0.01 (−0.02 to 0.04)	3 (2)	0 (0)	0.02 (−0.00 to 0.04)
Functional dependence	56 (46)	51 (45)	0.01 (−0.12 to 0.13)	82 (51)	25 (34)	0.16 (0.03–0.29)
Immobility	14 (12)	12 (11)	0.01 (−0.07 to 0.09)	20 (12)	6 (8)	0.04 (−0.04 to 0.12)
History of falls	28 (23)	14 (12)	0.10 (0.01–0.20)	29 (18)	13 (18)	0.00 (−0.10 to 0.10)
Visual impairment	98 (80)	96 (85)	−0.05 (−0.14 to 0.05)	137 (85)	57 (78)	0.06 (−0.04 to 0.17)
Hearing impairment	15 (12)	20 (18)	−0.05 (−0.14 to 0.04)	23 (14)	12 (16)	−0.02 (−0.12 to 0.08)
History of trauma	23 (19)	27 (24)	−0.05 (−0.15 to 0.05)	29 (18)	21 (9)	−0.01 (−0.22 to 0.01)
Comorbidities						
Dehydration	20 (16)	3 (3)	0.13 (0.06–0.20)	19 (12)	4 (6)	0.06 (−0.01 to 0.13)
Renal disease	7 (6)	5 (4)	0.01 (−0.04 to 0.07)	6 (4)	6 (8)	−0.04 (−0.11 to 0.02)
Liver disease	3 (2)	1 (1)	0.02 (−0.02 to 0.05)	3 (2)	1 (1)	0.00 (−0.02 to 0.04)
Depression	19 (16)	13 (12)	0.04 (−0.01 to 0.13)	22 (14)	10 (14)	−0.00 (−0.09 to 0.09)
Poly-pharmacy (>5 drugs)	48 (39)	36 (32)	0.07 (−0.05 to 0.19)	59 (37)	25 (34)	0.02 (−0.11 to 0.15)
Admission type						
Outpatient	61 (50)	43 (38)	−0.10 (−0.24 to 0.03)	70 (44)	34 (47)	−0.06 (−0.08 to 0.21)
23-h admission	31 (25)	38 (34)		46 (28)	23 (31)	
Inpatient	30 (25)	32 (28)		46 (28)	16 (22)	
Surgery type						
Cardiovascular/thoracic	4 (3)	4 (3.5)	−0.04 (−0.19 to 0.09)	4 (3)	4 (5)	0.02 (−0.12 to 0.18)
Ears/nose/throat	12 (10)	8 (7)		12 (7)	8 (11)	
General surgery	16 (13)	15 (13)		23 (14)	8 (11)	
Gynecological	6 (5)	4 (3.5)		4 (3)	6 (8)	
Neurosurgery	11 (9)	10 (9)		17 (10)	4 (6)	
Ophthalmology	17 (14)	11 (10)		25 (16)	3 (4)	
Orthopedics	41 (33)	51 (45)		58 (36)	34 (47)	
Plastic surgery	2 (2)	0 (0)		2 (1)	0 (0)	
Urology	13 (11)	10 (9)		17 (10)	6 (8)	

Data are presented as mean ± SD, median (first, third quartile), or *n* (% of column). Standardized differences reported as Hedges G for continuous data and as Cliff’s delta for ordinal and nominal data.

a Per protocol patients received no midazolam or long-acting opioids, and fentanyl within the guidelines of the ADP protocol. Patients that received scopolamine, famotidine, ondansetron, or dexamethasone were only considered ADP compliant if the drug was administered as part of ERAS protocol specific for their surgery.

**TABLE 2:** Operative and anesthesia data comparison between randomized no-delirium protocol and standard of care control groups.

Number of subjects	Intent to treat			Per protocol^c^		
	Control	Avoid delirium protocol	*P*	Non-protocol adherent	Protocol adherent	*P*
	122	113		162	73	
Primary anesthesia type						
General	66 (54)	68 (60)	0.49	87 (54)	47 (64)	0.21
Monitored anesthesia care	28 (23)	19 (17)		37 (23)	10 (14)	
Neuraxial/peripheral	28 (23)	26 (23)		38 (23)	11 (22)	
Intraoperative medication						
Opioid analgesics	86 (71)	89 (79)	0.18	121 (75)	54 (74)	0.91
Vasopressors	65 (53)	70 (62)	0.19	94 (58)	41 (56)	0.79
Dexamethasone	70 (58)	35 (31)	<0.001	88 (54)	17 (24)	<0.001
Ketamine	19 (16)	7 (6)	0.036	22 (14)	4 (6)	0.07
Midazolam	99 (82)	58 (51)	<0.001	102 (63)	0 (0)	<0.001
Ondansetron	76 (63)	73 (64)	0.79	117 (63)	47 (64)	0.83
Scopolamine	4 (2)	2 (1)	0.68	4 (3)	2 (2)	0.90
Famotidine	10 (8)	6 (5)	0.44	13 (8)	3 (4)	0.27
Intraoperative medication dosages						
Opioids						
MME^a^	5 (0, 15)	10 (3, 20)	0.039	10 (0, 20)	10 (0, 18)	0.89
MME/kg	0.15 (0.07, 0.34)	0.20 (0.10, 0.36)	0.14	0.16 (0.08, 0.34)	0.16 (0.08, 0.46)	0.59
Vasopressors						
VPE^b^	100 (0, 400)	160 (0, 410)	0.51	150 (0, 360)	160 (0, 523)	0.51
VPE/kg	5.2 (2.9, 10.3)	4.8 (2.5, 10.1)	0.77	4.3 (2.4, 8.9)	6.9 (3.5, 14.3)	0.03
Midazolam (mg/kg)	0.03 (0.01, 0.03)	0.03 (0.01, 0.03)	0.89	0.03 (0.01, 0.03)	–	–
Ketamine (mg/kg)	0.5 (0.3, 0.6)	0.5 (0.4, 1.0)	0.25	0.5 (0.4, 0.6)	0.4 (0.2, 0.6)	0.25
Dexamethasone (mg/kg)	0.1 (0.06, 0.13)	0.1 (0.06, 0.11)	0.59	0.1 (0.06, 0.12)	0.1 (0.05, 0.11)	0.41
Operative and surgical variables						
Intraoperative fluids (ml)	1,000 (600, 1,300)	1,000 (725, 1,500)	0.17	1,000 (650, 1,400)	1,000 (800, 1,500)	0.64
Hypotensive episode	81 (66)	79 (70)	0.58	105 (65)	55 (75)	0.13
Lowest SpO_2_	97 (95, 98)	97 (95, 99)	0.98	97 (95, age 98)	97 (95, 99)	0.19
Estimated blood loss (ml)	25 (5, 75)	40 (10, 100)	0.118	25 (5, 85)	25 (5, 100)	0.64
Duration of surgery (min)	76 (50, 121)	83 (57, 121)	0.47	81 (57, 124)	76 (46, 114)	0.11

Data are presented as median (first to third quartile) or *n* (%) of column.

a MME = IV morphine equivalents in patients that received an opioid analgesic preoperatively or intraoperatively.

b VPE = vasopressor phenylephrine equivalents where ephedrine to phenylephrine ratio was calculated as 80:1.

c Per protocol patients received no midazolam or long-acting opioids, and fentanyl within the guidelines of the ADP protocol. Patients that received scopolamine, famotidine, ondansetron, or dexamethasone were only considered ADP compliant if the drug was administered as part of ERAS protocol specific for their surgery.

**TABLE 3:** Results of delirium assessments in first 24 h following surgery.

	No delirium	Subsyndromal delirium	Incident delirium	*P*
Number of cases *n* (%)	211 (89.8)	16 (6.8)	8 (3.4)	
Time from PACU admission to first CAM assessment (min)	55 (36, 77)	81 (38, 179)	79 (59, 97)	0.010
Time from last administered ADP protocol drug to first CAM assessment (min)	84 (53, 129)	104 (39, 194)	115 (96, 156)	<0.001
NRS pain score at time of CAM assessment (0–10)	0 (0, 4)	3 (0, 6)	3 (2, 4)	0.08
RASS score at postoperative CAM-ICU assessment				
−3	0 (0)	0 (0)	4 (50)	<0.001
−2	1 (0.5)	6 (38)	0 (0)	
−1	1 (0.5	10 (67)	0 (0)	
0	209 (99)	0 (0)	3 (28)	
1	0 (0)	0 (0)	1 (12)	
Motoric state during first 24 h				
Hypoactive		15 (93)	7 (88)	1.0
Mixed		1 (7)	1 (12)	
Sleep (0 equals not restful, 10 equals extremely restful)	8 (5, 10)	5.5 (3, 9)	5.5 (5, 8)	0.22

Data are presented as *n* (% of total subjects) or median (first to third quartile).

**TABLE 4:** Univariable comparisons of subject characteristic, operative, and anesthesia factors in subjects with and without early (<24 h) delirium.

Number of subjects	No delirium	Early delirium	Difference (95% CI of the difference)	*P*
	211	24		
Age (year)	72 (68, 77)	70 (68, 74)	0.72 (0.44 to 1.17)	0.19
Female sex, *n* (%)	104 (49)	13 (54)	6 (−28 to 18)	0.81
Race, *n* (%)				
Caucasian	167 (79)	17 (71)	−8 (−12 to 29)	0.49
African American	28 (13)	4 (17)	4 (−21 to 14)	
Hispanic	12 (6)	3 (13)	7 (−22 to 9)	
Asian	4 (2)	0 (0)	−2 (−2 to 6)	
ASA physical status				
2	95 (45)	17 (71)	−26 (−48 to −3)	0.016
3	116 (55)	7 (29)	26 (3 to 48)	
BMI (kg/m^2^)	28.7 (25.5, 33.3)	29.2 (25.4, 33.3)	0.96 (0.56 to 1.56)	0.88
Work status				
Retired	147 (70)	18 (75)	5 (−15 to 26)	0.81
Working	64 (30)	6 (25)	−5 (−26 to 15)	
MMSE (0 to 30)	30 (29–30)	29.5 (28–30)	0.93 (0.55 to 1.56)	0.79
Risk factors				
History of delirium	3 (1)	0 (0)	−1 (−4 to 4)	0.56
Functional dependence	101 (48)	6 (25)	−23 (−2 to −44)	0.06
Immobility	22 (11)	4 (17)	6 (−11 to 24)	0.32
History of falls	36 (17)	6 (25)	8 (−12 to 28)	0.40
Visual impairment	177 (84)	17 (71)	−7 (−34 to 8)	0.15
Hearing impairment	31 (15)	4 (17)	2 (−19 to 16)	0.76
History of trauma	47 (22)	3 (12)	−10 (−26 to 7)	0.43
Comorbidities				
Dehydration	21 (10)	2 (8)	−2 (−15 to 12)	0.80
Renal disease	12 (5)	0 (0)	−5 (0 to −11)	0.62
Liver disease	4 (2)	0 (0)	−2 (−6 to 2)	0.50
Depression	27 (13)	5 (21)	8 (−11 to 27)	0.34
Poly pharmacy	75 (36)	9 (38)	2 (−20 to 24)	0.83
Primary anesthesia				
General	116 (55)	18 (75)	20 (−1 to 40)	0.032
Monitored anesthesia care	47 (22)	0 (0)	−22 (−30 to −14)	
Neuraxial/peripheral	48 (23)	6 (25)	2 (−23 to 18)	
Admission type				
Outpatient	100 (47)	4 (17)	−30 (−49 to −11)	0.002
23 h admission	62 (29)	7 (29)	0 (−22 to 22)	
Inpatient	49 (23)	13 (54)	31 (7 to 55)	
Surgery type				
Cardiovascular/thoracic	8 (4)	0 (0)	−4 (−8 to 1)	0.12
Ears/nose/throat	16 (8)	4 (17)	9 (−8 to 26)	
General surgery	29 (14)	2 (8)	−6 (−19 to 9)	
Gynecological	7 (3)	3 (13)	10 (−6 to 24)	
Neurosurgery	19 (9)	2 (8)	−1 (−13 to 11)	
Ophthalmology	28 (13)	0	−13 (−6 to −20)	
Orthopedics	80 (38)	12 (50)	12 (−11 to 35)	
Plastic surgery	2 (1)	0	−1 (−3 to 1)	
Urology	22 (10)	1 (4)	−6 (−17 to 5)	
Intraoperative medications				
Opioid analgesics	154 (73)	21 (88)	15 (−2 to 31)	0.14
Vasopressors	119 (56)	16 (67)	8 (−12 to 33)	0.40
Dexamethasone	91 (43)	14 (61)	18 (−8 to 38)	0.12
Ketamine	23 (11)	3 (13)	2 (−13 to 17)	0.73
Midazolam	144 (68)	13 (54)	−14 (−37 to 9)	0.18
Ondansetron	81 (38)	5 (21)	−17 (−37 to 2)	0.12
Scopolamine	6 (3)	0 (0)	−3 (−7 to 2)	0.40
Famotidine	16 (7)	0 (0)	−7 (−13 to 2)	0.38
MME	7.5 (0, 15)	18.8 (8.2, 25)	2.1 (1.3 to 3.5)	0.004
VPE	155 (0, 415)	200 (0, 400)	1.1 (0.69 to 1.80)	0.66
Operative and Surgical Variables				
Intraoperative fluids (ml)	1,000 (660–1,400)	1,300 (900–1,950)	1.9 (1.2 to 3.2)	0.011
Intraoperative hypotension	144 (68)	16 (67)	5 (−34 to 45)	0.87
Lowest SpO_2_	97 (95, 99)	98 (95, 99)	1.2 (0.76 to 1.99)	0.41
Estimated blood loss (ml)	25 (5, 100)	50 (28, 50)	1.6 (0.97 to 2.59)	0.07
Duration of surgery (min)	78 (50, 117)	108 (74, 145)	1.9 (1.2 to 3.2)	0.010
Pain in first hour in the PACU	0 (0–4)	3 (0–6)	1.7 (1.0 to 2.7)	0.046

Data are presented as *n* (% of column) or median (first, third quartile).

## Data Availability

The raw data supporting the conclusions of this article will be made available by the authors, without undue reservation.
